# Multimodal deep learning approaches for precision oncology: a comprehensive review

**DOI:** 10.1093/bib/bbae699

**Published:** 2025-01-05

**Authors:** Huan Yang, Minglei Yang, Jiani Chen, Guocong Yao, Quan Zou, Linpei Jia

**Affiliations:** Yangtze Delta Region Institute (Quzhou), University of Electronic Science and Technology of China, Chengdian Road, Kecheng District, Quzhou 324000, Zhejiang, China; Department of Pathology, The First Affiliated Hospital of Zhengzhou University, Jianshe Dong Road, Erqi District, Zhengzhou 450052, Henan, China; Yangtze Delta Region Institute (Quzhou), University of Electronic Science and Technology of China, Chengdian Road, Kecheng District, Quzhou 324000, Zhejiang, China; School of Opto-electronic and Communication Engineering, Xiamen University of Technology, Ligong Road, Jimei District, Xiamen 361024, Fujian, China; Yangtze Delta Region Institute (Quzhou), University of Electronic Science and Technology of China, Chengdian Road, Kecheng District, Quzhou 324000, Zhejiang, China; School of Computer and Information Engineering, Henan University, Jinming Avenue, Longting District, Kaifeng 475001, Henan, China; Yangtze Delta Region Institute (Quzhou), University of Electronic Science and Technology of China, Chengdian Road, Kecheng District, Quzhou 324000, Zhejiang, China; Institute of Fundamental and Frontier Sciences, University of Electronic Science and Technology of China, Section2, North Jianshe Road, Chenghua District, Chengdu 610054, Sichuan, China; Department of Nephrology, Xuanwu Hospital, Capital Medical University, Changchun Street, Xicheng District, Beijing 100053, China

**Keywords:** multimodal, deep learning, cancer, integration

## Abstract

The burgeoning accumulation of large-scale biomedical data in oncology, alongside significant strides in deep learning (DL) technologies, has established multimodal DL (MDL) as a cornerstone of precision oncology. This review provides an overview of MDL applications in this field, based on an extensive literature survey. In total, 651 articles published before September 2024 are included. We first outline publicly available multimodal datasets that support cancer research. Then, we discuss key DL training methods, data representation techniques, and fusion strategies for integrating multimodal data. The review also examines MDL applications in tumor segmentation, detection, diagnosis, prognosis, treatment selection, and therapy response monitoring. Finally, we critically assess the limitations of current approaches and propose directions for future research. By synthesizing current progress and identifying challenges, this review aims to guide future efforts in leveraging MDL to advance precision oncology.

## Introduction

### Precision oncology

Cancer remains one of the foremost causes of mortality globally. The 2020 report from the International Agency for Research on Cancer identified ~18.1 million new cancer cases and 9.6 million cancer-related deaths across 185 countries, both figures rising alarmingly [1]. In the USA, the economic burden of cancer was estimated at ~$124.5 billion in 2010, with projections rising to $157.8 billion by 2020 [2]. The emergence of novel cancer therapies, such as targeted therapies and immunotherapies, underscores the potential for curative outcomes through early detection and effective treatment [3, 4]. Consequently, early diagnosis, precise tumor classification, and personalized treatment are critical for improving survival rates, enhancing quality of life for cancer patients, and alleviating the societal economic burden.

### DL in precision oncology

Over the past two decades, advances in computing technology has propelled deep learning (DL) to the forefront of precision oncology. For instance, DL models used in low-dose computed tomography (CT) lung cancer screening have successfully reduced the pool of candidates while maintaining high inclusion rates and positive predictive values [5]. Natural language processing (NLP) techniques are increasingly applied to extract valuable insights from electronic health records (EHRs), aiding clinicians in decision-making [6]. DL has demonstrated exceptional performance in tasks such as biological sequence classification [7] and cancer subtyping [8], with artificial intelligence (AI) systems even surpassing human experts in certain diagnostic areas [9]. Moreover, DL has shown promise in predicting cancer prognosis. For example, a DL-based model leveraging pathological biomarkers was able to stratify colorectal cancer (CRC) patients into distinct prognostic groups, minimizing overtreatment in low-risk patients and identifying those who would benefit from more aggressive therapies [10]. DL also holds potential in personalized treatment planning and predicting therapeutic responses [11].

While these unimodal DL applications have achieved significant success, the rapid advancement of computing and biomedical technologies, along with the explosive growth of clinical data, highlights the urgent need for integrated multimodal data analysis to fully harness clinical information and gain deeper insights into cancer mechanisms.

### Clinical value of multimodal fusion analysis

Vast amounts of multimodal data are generated throughout the clinical process of cancer care. Multimodal analysis techniques leverage the unique characteristics of each modality to develop models that offer a more comprehensive understanding and reasoning. This approach closely aligns with real-world clinical practices, particularly for complex diseases. Common multimodal fusion models include the integration of various medical imaging types, such as whole slide image (WSI) and CT [12], as well as diverse magnetic resonance imaging (MRI) sequences [13], fostering opportunities for innovative fusion strategies. Additionally, the amalgamation of multi-omics data and the fusion of molecular omics with imaging data further exemplifies this trend [14, 15]. Cross-modal fusion that encompasses imaging, molecular, and clinical data represents advanced stages of multimodal analysis [16].

Numerous studies have shown that multimodal methods outperforms single-modality approaches in specific tasks [17, 18]. Nevertheless, designing effective fusion methods presents several challenges, including the high-dimensional nature of multimodal data, issues with data incompleteness and modality imbalance, and the need for real-time processing. Moreover, uncovering the biological significance of multimodal features remains a significant hurdle.

Existing reviews have highlighted key DL applications in cancer diagnosis, prognosis, and treatment selection [19, 20], with many focusing on specific data types or cancer categories [21–24], exploring the taxonomy of MDL models for biomedical data integration [25], or discussing DL-based multimodal feature fusion for identifying cancer biomarkers [26]. However, these reviews typically focus on single-modal data or are limited to multi-omics data, without offering a comprehensive overview of cross-scale multimodal data fusion. Consequently, a thorough review of MDL methods across the entire precision oncology continuum is still lacking.

**Figure 1:**
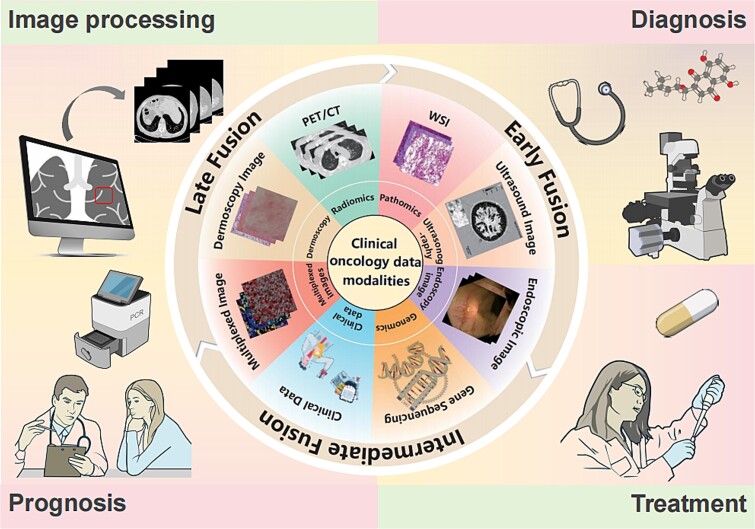
Scope of this review. Multimodal oncology data, DL techniques, and corresponding MDL applications for precision medicine.

Given the rapid expansion of medical multimodal data and the swift evolution of MDL technologies, this paper aims to survey various modalities involved in precision oncology and the cutting-edge MDL models employed for data integration, thereby establishing a paradigm for the effective utilization of big data in cancer management (Fig. 1).

### Structure of the work

The paper is structured as follows: it begins with a discussion of literature search strategies, followed by an overview of publicly available multimodal oncology datasets and an introduction to key DL technologies. Next, we examine modality representation and fusion techniques, survey MDL applications in precision oncology, and explore the opportunities and challenges in integrating oncology big data. The paper concludes with a forward-looking perspective on future developments.

### Search methods

A systematic search was conducted in September 2024 across PubMed, MEDLINE, and Web of Science Core Collection for peer-reviewed articles published in English, with no date restrictions. Search terms included medical topics (e.g. cancer, tumors, lesions), methodologies (e.g. deep learning, artificial intelligence, convolutional neural networks, machine learning), and data types (e.g. multimodal, multi-omics, data fusion). Two independent researchers performed the search to ensure accuracy; disagreements were resolved by a third investigator with domain expertise. Initial screening was based on titles and abstracts, followed by full-text review. Only original research involving human subjects and with full-text availability was included, excluding reviews, posters, and comments. A total of 651 articles met the inclusion criteria and were analyzed, with selected studies discussed to provide insights into MDL applications in oncology.

### Public multimodal oncology resources

Cancer is a highly complex, heterogeneous biological process, requiring diverse data sources for accurate diagnosis, treatment, and prognosis. Commonly used data types—either individually or in combination—include radiomics, pathomics, acoustic and endoscopic imaging, genomics, clinical data, dermoscopy, multimodal data, and emerging real-world data (Supplementary Materials 1). Here, we summarized credible publicly available multimodal oncology resources and representative MDL studies utilizing these datasets (Table 1) for the readers’ convenience.

**Table 1:** Public oncology resources for MDL algorithm development

Resource	Cancer	Modality	URL	Representative studies
TCGA	Pan-cancer	Histopathology, multi-omics, clinical data	https://portal.gdc.cancer.gov/	[14, 27–30]
TCIA	Pan-cancer	Histopathology, radiology, MR, US, clinical data	https://www.cancerimagingarchive.net/	[31, 32]
Lung-CLiP	Lung	Clinical data, SNV, CNV	https://doi.org/10.1038/s41586-020-2140-0; http://clip.stanford.edu	[33]
DELFI	Lung	Clinical data, cfDNA	https://doi.org/10.1016/j.chest.2023.04.033; EDA accession No. EGAS00001005340	[34]
ASVM	Pan-cancer	Clinical data, cfDNA, CNV	https://github.com/ElaineLIU-920/ASVM-for-Early-Cancer-Detection	[35]
HAM10000	Skin	Dermoscopic image, clinical data	https://doi.org/10.7910/DVN/DBW86T; https://isic-archive.com/api/v1	[36]

*Note*: SNV, single-nucleotide variation; CNV, copy number variation; cfDNA, cell-free DNA.

Notably, The Cancer Genome Atlas (TCGA) (https://portal.gdc.cancer.gov/) and The Cancer Imaging Archive (TCIA) (https://www.cancerimagingarchive.net/) are extensive databases encompassing thousands of samples across various cancer types and medical centers. They provide rich multimodal data and analytical tools essential for cancer research. In addition to large-scale public databases, several specialized multimodal datasets are available. For instance, Lung-CLiP (Lung Cancer Likelihood in Plasma) provided clinical, demographic, and genome-wide single-nucleotide variation (SNV) and copy number variation (CNV) data for lung cancer cases, as well as codes for reproduction of corresponding results [33]. DNA Evaluation of Fragments for Early Interception (DELFI) offers cell-free DNA (cfDNA) fragmentation profiles and clinical data for 296 lung cancer patients [34]. Adaptive Support Vector Machine (ASVM) integrates cfDNA fragmentome, CNVs, and clinical data for 423 patients across eight cancer types [35]. The HAM10000 dataset consists of 10 015 multicenter dermatoscopic images with corresponding clinical data aimed at improving melanoma detection [36]. These resources are invaluable for developing and evaluating MDL algorithms for patient profiling.

### Overview of DL techniques

DL algorithms leverage modular structures to perform complex functions. For prevalent DL architectures that are applicable across diverse data types, please see Supplementary Materials 2. The table of abbreviations and their full forms can be found in Supplementary Materials 3. Due to privacy and other reasons, obtaining medical data often faces distinct kinds of limitations. Missing modalities and labels are common in multimodal datasets, which contrasts sharply with DL models’ eagerness for large amounts of labeled data. Fortunately, several techniques have shown promise to reduce the reliance on extensive data labeling while maintaining model performance and data security.

#### Transfer learning

Transfer learning (TL) has emerged as a powerful tool in the field of DL-based medical data analysis [37]. By delivering knowledge from one domain to another, TL facilitates the resolution of analogous tasks. The common attributes found in natural images, such as colors, edges, corners, and textures, can aid in medical image tasks like registration, segmentation, and classification. By maintaining most of the pretrained model weights and just fine-tuning the last layers, both generalized and domain-specific features are learned, thus saving much annotated data, time, and computational resources.

#### Federated learning

Federated learning (FL) is an innovative approach to safeguarding the privacy and security of medical data. It allows multiple participants (referred to as clients) to collaboratively train a global model without sharing their local data [38]. In this framework, a central server coordinates multiple training rounds to produce the final global model. At the beginning of each round, the server distributes the current global model to all clients. Each client then trains the model on their local data, updates it, and returns the modified model to the server. The server aggregates these updates to enhance the global model, thus completing one training cycle. Throughout this process, participants’ data remain on their devices, and only encrypted model updates are exchanged with the server, ensuring data confidentiality.

### Supervise or not?

The efficacy of DL models hinges on the quality and quantity of training data. In precision oncology, four primary learning paradigms—supervised, weakly supervised, self-supervised, and unsupervised learning—have emerged as pivotal techniques. While each method possesses distinct characteristics, they are interconnected and can be complementary in certain applications.

#### Supervised learning

Supervised learning (SL) involves training models on labeled datasets, where each data point is associated with a corresponding target variable. The model learns to map input features to output labels, minimizing the discrepancy between predicted and actual values. SL excels in predictive accuracy, making it widely used for tasks such as classifying tumor subtypes and predicting patient outcomes [39, 40]. However, SL requires substantial labeled datasets, which can be challenging to obtain in healthcare, and it assumes a specific data distribution, potentially limiting generalization ability to unseen data.

#### Weakly supervised learning

Weakly supervised learning (WSL) addresses the scarcity of labeled data by leveraging partially labeled or noisy datasets. A prominent technique is multiple instance learning, which operates on bags of instances where only the bag is labeled [41]. The model learns to identify patterns within instances to make predictions for the entire bag. WSL can also use labeling functions to create training sets. However, weakly supervised labels are often less accurate than those from human experts, necessitating careful consideration.

#### Self-supervised learning

Self-supervised learning (SSL) allows the model to generate its own labels from the data. Users create a pretext task related to the primary task of interest. By solving this pretext task, pseudo-labels are produced based on specific input attributes, enabling the model to learn representations transferable to the primary task, even with limited labeled data [42]. SSL is especially useful when labeled data are scarce or costly to acquire; however, the design of the pretext task is crucial for ensuring the relevance of the learned representations.

#### Unsupervised learning

Unsupervised learning (USL) operates on unlabeled data, identifying patterns and structures without explicit supervision. Statistical methods are employed to uncover underlying relationships. Techniques such as clustering analysis, dimensionality reduction, and association rule learning exemplify USL [43–45]. USL offers the advantage of discovering novel knowledge without relying on labeled data. However, its results can be non-unique and less interpretable, making it less suitable for applications where accuracy is paramount.

The choice of learning paradigm in precision oncology depends on the specific application, data availability and quality, and the desired level of accuracy and interpretability. SL is ideal for tasks with ample labeled data and clear target variables, while WSL is beneficial when data are limited or noisy. SSL is effective for pretraining models on large unlabeled datasets, and USL is valuable for exploratory data analysis. By understanding the strengths and limitations of each paradigm, researchers can select the most suitable approach for their specific research questions.

### Integration techniques for multimodal data with DL

Multimodal modeling addresses the complexities of unstructured multimodal data, such as images, text, and omics data. It faces two primary challenges: first, effectively representing data from each modality; and second, integrating data from diverse modalities. This section provides an overview of current technical approaches to these challenges.

#### Multimodal representation

Multimodal representation involves extracting semantic information from diverse data forms into real-valued vectors. Medical data encompass structured, semi-structured, and unstructured formats. Effective data representation methods are vital for revealing relational insights, thereby facilitating accurate computer-aided diagnosis and prognosis. This representation can be categorized into unimodal and cross-modal approaches, as detailed below.

#### Unimodal representation

Unimodal representation, or marginal representation, focuses on distilling key information from a single modality through various encoding techniques. For textual data, exemplified by EHRs, word embeddings transform phrases into dense vectors that capture their semantic meanings, ensuring similar phrases are closely clustered in a low-dimensional feature space. For imaging modalities like CT, MRI, and WSI, data are converted into 2D or 3D pixel matrices, suitable for convolutional neural networks (CNNs). In ultrasound, endoscopic, and other video data, individual frames are segmented and encoded similarly to static images. For genomic and transcriptomic data, one-hot encoding is commonly employed.

#### Cross-modal representation

Cross-modal representation, or joint representation, integrates features from multiple modalities, capturing complementary, redundant, or cooperative information. Canonical correlation analysis (CCA) is a traditional method for cross-modal information representation, mapping multimodal data—such as images and text—into a shared latent space by identifying linear combinations of multidimensional variables [46]. While CCA enhances multimodal model performance, its linear assumptions and sensitivity to noise constrain its effectiveness. Recent advancements focus on multimodal interaction mining and model efficiency. For example, Zhen *et al*. developed a spectral hashing coding strategy for rapid cross-modal retrieval by employing spectral analysis of various modalities [47]. Cheerla *et al*. implemented an attention network to extract cross-modal features from gene expression data, pathological images, and clinical information, projecting them into a joint feature space for representation learning [48]. Zhao *et al*. proposed a hierarchical attention encoder-reinforced decoder network to generate natural language answers in open-ended video question answering [49].

Despite these advancements, current research inadequately addresses interference and adverse effects from modality-specific information irrelevant to target tasks. Additionally, existing encoding methods, often derived from natural language or image processing, may be overly simplistic for the specialized context of medical data, leading to complex, redundant structures and low parameter efficiency in developing multimodal learning frameworks.

### Multimodal fusion

**Figure 2:**
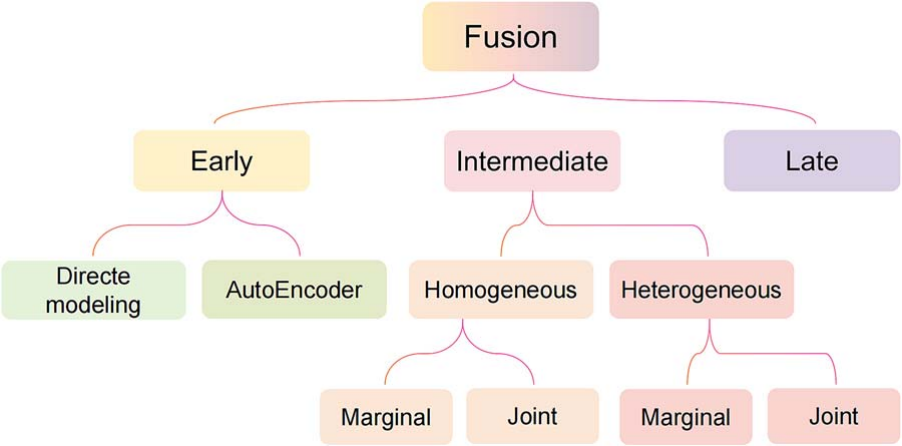
Taxonomy of multimodal fusion strategies.

Multimodal feature fusion strategies can be broadly categorized into three types: data-level fusion, model-based fusion, and decision-level fusion. When classified by the stage at which fusion occurs, these correspond to early fusion, intermediate fusion, and late fusion, respectively (Fig. 2).

#### Early fusion

**Figure 3:**
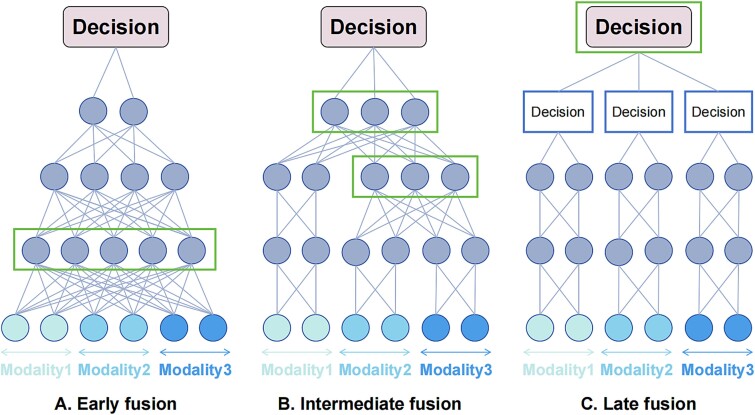
Schematic diagram of (A) early fusion, (B) intermediate fusion, and (C) late fusion. The rectangular box in each section represents the fusion stage of multimodal features.

Early fusion is the most straightforward approach for integrating multimodal data, wherein features from diverse modalities are concatenated and directly input into a DL model (Fig. 3A). This technique treats the resulting vector as a unimodal input, preserving the original model architecture. Joint representations of multimodal inputs are learned directly, bypassing explicit marginal representations. Early fusion can be further divided into two categories: direct modeling and AutoEncoder (AE) methods.

In direct modeling, multimodal inputs are processed similarly to unimodal inputs. For example, Misra *et al*. developed a multimodal fusion framework to classify benign and malignant breast lesions by processing brightness-mode (B-mode) and strain-elastography-mode (SE-mode) ultrasound images through separate CNNs for feature extraction, which were subsequently ensembled using another CNN model [50]. AE methods initially learn lower-dimensional joint representations, which are then employed for further supervised or unsupervised modeling. For instance, Allesøe *et al*. utilized a Variational AutoEncoder (VAE) model to integrate multi-omics data, identifying drug–omics associations across multimodal datasets for type 2 diabetes patients [51].

While early fusion effectively captures low-level cross-modal relationships without requiring marginal representation extraction, it may struggle to discern high-level relationships and is sensitive to differences in the sampling rates of various modalities.

#### Intermediate fusion

Intermediate fusion involves initially learning each modality independently before integrating them within a MDL framework (Fig. 3B). This method focuses on generating marginal representations prior to fusion, allowing for greater flexibility. Intermediate fusion can be categorized into homogeneous fusion and heterogeneous fusion based on the networks used for marginal representation (Fig. 2). In homogeneous fusion, identical neural networks are employed to learn marginal representations across modalities, making it suitable for homogeneous modalities. Heterogeneous fusion is applied when modalities differ significantly, necessitating distinct neural networks for representation learning. Furthermore, both fusion types can be divided into marginal and joint categories based on representation handling. Marginal intermediate fusion concatenates learned marginal representations as inputs to fusion layers, while joint intermediate fusion encodes more abstract features from multiple modalities prior to integration.

In marginal homogeneous intermediate fusion, identical neural networks learn marginal representations, which are later combined for decision-making. For example, Gu *et al*. employed a 3D U-Net to encode positron emission tomography (PET) and CT images as separate channels, integrating them during the decoding phase to generate pulmonary perfusion images [52].

Marginal heterogeneous intermediate fusion uses distinct network types for different modalities. The Pathomic Fusion model, for instance, extracted histological features via CNNs or graph convolutional neural network, while genomic features were captured using a feed-forward network. These multimodal features were then fused through a gating-based attention mechanism combined with the Kronecker product function [14].

Joint homogeneous fusion begins with concatenating marginal representations, followed by joint representation learning from this composite. For example, Yuan *et al*. constructed two identical convolutional–long short-term memory (Conv-LSTM) encoders to extract features from PET and CT, respectively, and these features were concatenated and transformed by a LSTM module for the sample [53].

Joint heterogeneous intermediate fusion employs different networks for each modality, subsequently deriving joint representations from concatenated marginal representations. Hu *et al*. illustrated this by using a ResNet-Trans network for CT features and a graph to model relationships between clinical and imaging features, learning joint representations with a graph neural network for lymph node metastasis prediction [54].

In summary, intermediate fusion strategies offer significant flexibility in determining optimal fusion depth and sequence, potentially revealing more accurate relationships between modalities. However, implementing intermediate fusion requires considerable computational expertise and resources.

#### Late fusion

Late fusion, inspired by ensemble classification, consolidates predictions from individual sub-models trained on distinct data modalities to make a final decision (Fig. 3C). This can be accomplished through various methods, including voting, averaging, or meta-learning. For example, Saikia *et al*. compared majority voting and weighted voting approaches for predicting human papillomavirus status using PET-CT images [31]. Sedghi *et al*. improved prostate cancer detection by averaging outputs from temporal enhanced ultrasound and MRI-based U-Nets [55]. Qiu *et al*. introduced an attention-based late fusion strategy to integrate complementary information from WSIs and CNVs for lung cancer classification [27].

While late fusion facilitates comprehensive marginal representation learning from unimodal models, the reduced interaction between modalities may lead to irrelevant multimodal features and complicate model interpretation.

Each fusion strategy has unique advantages and limitations. The optimal approach depends on various factors, including data heterogeneity, researcher intuition, biological implications, the presence of missing values or noise, experimental evidence, computational resources, or a combination of these elements.

### MDL applications in precision oncology

The integration of AI at various stages can correlate clinical laboratory tests and examination data with oncological phenotypes. The adaptability of clinical tasks involving multimodal data varies across different contexts. This section delves into cutting-edge MDL applications in cancer management, emphasizing image analysis, cancer detection, diagnosis, prognosis, and treatment.

#### Image registration and segmentation

Image processing represents a core application of ML in oncology, with key tasks including multimodal image registration and segmentation. The integration of PET and CT images for lesion identification and tumor volume delineation is prevalent in clinical practice, yet it remains challenging. Gu *et al*. utilized a 3D U-Net architecture, leveraging PET and CT images as dual channels within a marginally homogeneous intermediate fusion strategy, significantly enhancing the accuracy of pulmonary perfusion volume quantification compared to methods relying solely on metabolic data [52]. The complexity of understanding spatial correspondences increases when input modalities exhibit substantial discrepancies in appearance. To mitigate this, Song *et al*. proposed a contrastive learning–based cross-modal attention block that correlates features extracted from transrectal ultrasound (TRUS) and MRI. These correlations were integrated into a deep registrator for modality fusion and rigid image registration [56]. Additionally, Haque *et al*. correlated hematoxylin and eosin–stained WSIs with mass spectrometry imaging (MSI) data to facilitate modality translation, aiming to predict prostate cancer directly from WSIs [57].

Segmentation is another classical challenge in image analysis, critical for accurate diagnosis, therapeutic selection, and efficacy evaluation. However, segmenting soft tissue tumors, particularly brain tumors, poses significant challenges due to their complex physiological structures. Zhao *et al*. introduced an innovative glioma tumor segmentation method that integrates fully convolutional networks (FCNs) and recurrent neural networks (RNNs) within a unified framework, achieving segmentation results characterized by both appearance and spatial consistency. They trained three segmentation models using 2D MRI patches from axial, coronal, and sagittal views, merging results through a voting-based fusion strategy [58]. Beyond tissue or organ segmentation, cell segmentation is fundamental for various downstream biomedical applications, including tumor microenvironment exploration and spatial transcriptomics analysis. In a challenge aimed at advancing universal cell segmentation algorithms across diverse platforms and modalities [59], Lee *et al*. employed SegFormer and a multiscale attention network as the encoder and decoder, achieving superior performance in both cell recognition and differentiation across multiple modalities [60]. Relevant research mentioned in this section is summarized in Table 2 for further inspection.

**Table 2:** Representative studies focus on multimodal image process

Topic	Study (year)	Cancer	Modality	Backbone	Learning method	Dataset	Fusion strategy	Code availability	Data availability
Registration	Song *et al*. (2023) [56]	Prostate	MRI, TRUS	CNN, attention	Supervised	662 patients	Intermediate	https://github.com/DIAL-RPI/ Attention-Reg	Partial available
Registration	Haque *et al*. (2023) [57]	Prostate	MSI, WSI	CNN	Supervised	5 patients	Early	https://github.com/inzamam1190/HEtoMALDI	Need request
Registration and segmentation	Gu *et al*. (2023) [52]	Esophagus, lung	PET-CT	FCN	Supervised	53 patients	Early	No	Need request
Segmentation	Lee *et al*. (2023) [60]	Various cancers	Multimodal microscopy images	Transformer	Semi-supervised	7242 images	Early	https://github.com/Lee-Gihun/MEDIAR	Yes
Segmentation	Zhao *et al*. (2018) [58]	Glioma	Multimodal MRI	FCN, RNN	Supervised	465 patients	Late	No	Yes

*Note*: CNN, convolutional neural network; TRUS, transrectal ultrasound; MSI, mass spectrometry image; WSI, whole slide image; FCN, fully convolutional network; RNN, recurrent neural network.

#### Cancer detection, diagnosis, and metastasis prediction

Early detection is paramount for timely treatment and favorable prognosis. Currently, MDL methods offer clinicians with unprecedented opportunities to comprehensively assess patients’ tumor status (Table 3). For instance, Li *et al*. proposed a VAE-based framework that integrates single-cell multimodal data, utilizing SNV features alongside gene expression characteristics to classify tumor cells [15]. Liu *et al*. introduced AutoCancer, which integrates feature selection, neural architecture search, and hyperparameter optimization, demonstrating strong performance in cancer detection using heterogeneous liquid biopsy data [62].

**Table 3:** Representative studies focus on cancer detection and diagnosis

Topic	Study (year)	Cancer	Modality	Backbone	Learning method	Dataset	Fusion strategy	Code availability	Data availability
Cancer detection	Li *et al*. (2024) [15]	Pan-cancer	SNV, gene expression	VAE	Unsupervised	30 samples	Intermediate	No	Yes
Cancer detection	Wang *et al*. (2021) [61]	Melanoma	Dermoscopic image, clinical data	CNN	Supervised	1011 patients	Late	No	Yes
Cancer detection	Liu *et al*. (2024) [62]	Pan-cancer	SNV, CNV, clinical data	Transformer	Supervised	964 patients	Early	https://github.com/ElaineLIU-920/AutoCancer.git	Yes
Diagnosis	Gao *et al*. (2021) [63]	Hepatoma	Multiphase CECT, clinical data	CNN, RNN	Supervised	723 patients	Intermediate	https://github.com/ruitian-olivia/STIC-model	Need request
Diagnosis	Park *et al*. (2021) [64]	Lung	CT, PET-CT, clinical data	CNN	Supervised	359 patients	Early	No	No
Diagnosis	Khan *et al*. (2023) [12]	Liver	WSI, CT	CNN	Supervised	248 patients	Early	No	No
Diagnosis	Wu *et al*. (2024) [65]	Breast	Multimodal ultrasound	CNN	Supervised	733 patients	Late	Need request	Need request
Diagnosis	Wang *et al*. (2024) [66]	Ovarian	Ultrasound, serum indicator, clinical data	CNN	Supervised	1054 patients	Intermediate	https://data.mendeley.com/datasets/f5p4h32p46/2	Need request
Diagnosis	Xiang *et al*. (2024) [67]	Ovarian	Multimodal ultrasound, radiomics score, clinical	CNN	Supervised	724 female patients	Intermediate	https://github.com/Xiao-OMG/OvcaFinder; https://doi.org/10.5281/zenodo.10691378	Need request
Diagnosis	Du *et al*. (2023) [68]	Gastric	Multimodal endoscopy	CNN	Supervised	3449 images	Early, intermediate, late	No	Need request
Diagnosis	Wang *et al*. (2022) [69]	Skin	Dermoscopic image, clinical data	CNN	Supervised	10 015 images	Intermediate	No	Yes
Diagnosis	Qiu *et al*. (2023) [27]	Glioma, lung	WSI, CNV	GNN, SNN	Weakly supervised	683 glioma patients; 987 lung cancer patients	Intermediate	No	Yes
Metastasis prediction	Hu *et al*. (2023) [54]	Lung	CT, clinical data	ResNet, Transformer, GNN	Supervised	681 patients	Intermediate	No	No
Metastasis prediction	Zhong *et al*. (2023) [70]	Lung	PET-CT	CNN	Supervised	3265 patients	Early	https://github.com/zhongthoracic/DLNMS	Need request

*Note*: SNV, single-nucleotide variation; VAE, variational autoencoder; CNN, convolutional neural network; CNV, copy number variation; CECT, contrast-enhanced CT; RNN, recurrent neural network; GNN, graph neural network; SNN, self-normalizing networks.

Precision in tumor diagnosis is a vital area for medical AI applications. Gao *et al*. employed CNN and RNN as encoders for multiphase contrast-enhanced CT (CECT) and corresponding clinical data. These feature sets were concatenated to differentiate malignant hepatic tumors [63]. Park *et al*. found that incorporating metadata, such as the maximum value of the standard uptake (SUVmax) and lesion size, enhanced the performance of unimodal CT and PET models [64]. Khan *et al*. combined CT features with pathological features using fully connected layers to classify liver cancer variants [12]. Wu *et al*. developed a clinically aligned platform for grading ductal carcinoma *in situ*, treating each angle of ultrasound images as a separate modality and deriving final predictions through max pooling across all angles [65]. Wang *et al*. constructed multiple models for ovarian lesion classification with ultrasound, menopausal status, and serum data. Their trimodal model achieved superior predictive accuracy compared to both dual-modality and single-modal approaches [66]. Similarly, OvcaFinder was created for ovarian cancer identification, integrating ultrasound images, radiological scores, and clinical variables [67]. Du *et al*. aimed to enhance real-time gastric neoplasm diagnosis by constructing and comparing five models based on multimodal endoscopy data. Their results indicated that the multimodal model using the immediate fusion strategy yielded the best performance [68]. Carrillo-Perez *et al*. presented a late fusion model combining histology and RNA-Seq data for lung cancer subtyping, demonstrating that this integrative classification approach outperformed reliance on unimodal data [28]. Qiu *et al*. integrated pathology and genomics data for cancer classification. Their weakly supervised design and hierarchical fusion strategy maximized the utility of WSI labels and facilitate efficient multimodal interactions [27]. Wang *et al*. employed a late fusion approach to integrate clinical and dermoscopy images for malignant melanoma detection [61]. Another study combined skin lesion images with patient clinical variables, constructing a multiclass classification model [69].

Nodal involvement and distant metastasis are critical for definitive diagnosis, therapeutic decision-making, and prognosis in cancer patients. Hu *et al*. integrated CT and clinical features using a ResNet-Trans and graph neural network (GNN)–based framework, showcasing promise in predicting lymph node metastasis (LNM) in non–small cell lung cancer (NSCLC) patients [54]. Zhong *et al*. developed a PET-CT-based cross-modal biomarker to predict occult nodal metastasis in early-stage NSCLC patients, indicating the superiority of their multimodal model over single-modal approaches [70].

Overall, tumor detection, diagnosis, and metastasis prediction involve a diverse array of tumor data modalities, encompassing both the fusion of similar modalities and the integration of highly heterogeneous modalities. CNNs are commonly employed in diagnostic models, where supervised learning techniques prevail. Intermediate fusion strategies are frequently utilized, with comparative studies indicating that intermediate fusion often surpasses early and late fusion in efficacy. Moreover, the interpretability of features in ML models remains a crucial factor influencing their potential for clinical translation.

#### Prognosis prediction

The ability to predict recurrence and survival time in cancer patients is crucial for selecting and optimizing treatment regimens, particularly in advanced-stage tumors. Enhancing prognosis prediction through the integration of multiple early tumor indicators could significantly improve the accuracy of clinical interventions, leading to better patient outcomes and reduced waste of medical resources.

Recently, MDL has garnered significant attention in tumor prognosis prediction (Table 4). For instance, Li *et al*. developed a two-stage framework that decouples multimodal feature representation from the fusion process, demonstrating advantages in predicting the postoperative efficacy of cytoreductive surgery for CRC [71]. Miao *et al*. integrated radiomic features with clinical information, revealing relationships between body composition changes, breast cancer metastasis, and survival [72]. In another study, Fu *et al*. introduced a heterogeneous graph-based MDL method that encodes both the spatial phenotypes from imaging mass cytometry (IMC) and clinical variables, achieving remarkable performance in prognosis prediction across two public datasets [73]. Malnutrition is also a critical factor in cancer prognosis; Huang *et al*. combined non-enhanced CT features with clinical predictors to develop models for assessing nutritional status in gastric cancer, thereby enhancing preoperative survival risk prediction [77]. Huang *et al*. constructed an ensemble model based on EfficientNet-B4, utilizing both PET and CT data to predict progression in lung malignancies and overall survival (OS). Their findings indicated that this dual-modality model outperformed the PET-only model in accuracy and sensitivity, although no significant differences were observed compared to the CT-only model [78]. FL presents a promising solution to the challenges posed by small medical datasets and stringent privacy concerns. For instance, FedSurv is an asynchronous FL framework that employs a combination of PET and clinical features to predict survival time for NSCLC patients [75].

**Table 4:** Representative studies focus on cancer prognosis

Topic	Study (year)	Cancer	Modality	Backbone	Learning method	Dataset	Fusion strategy	Code availability	Data availability
Prognosis prediction	Li *et al*. (2023) [71]	CRC	CT, clinical data	CNN	Supervised	185 patients	Early	https://github.com/cchencan/DeAF	No
Metastasis and survival prediction	Miao *et al*. (2022) [72]	Breast	CT, clinical data	CNN	Unsupervised	431 patients	Early	https://github.com/HotHeaven233/DeepLearning-Radiomics-Research-in-Breast-Cancer	Yes
Survival prediction	Fu *et al*. (2023) [73]	Breast	IMC, clinical data	GNN, CNN	Unsupervised	741 patients	Intermediate	https://github.com/xhelenfu/DMGN_Survival_Prediction	Yes
Survival prediction	Huang *et al*. (2024) [74]	Gastric	CT, clinical data	CNN	Supervised	312 patients	Late	No	No
Survival prediction	Vo *et al*. (2024) [75]	NSCLC	PET, clinical data	CNN	Supervised	2898 patients	Early	No	No
Prognosis prediction	Yuan *et al*. (2023) [53]	DLBCL	PET-CT	Conv-LSTM	Supervised	249 patients	Early, intermediate	https://github.com/cyuan-sjtu/MDL-model	Need request
Prognosis prediction	Volinsky-Fremond *et al*. (2024) [29]	Endometrial	WSI, clinical data	ViT	Self-supervised, supervised	2072 patients	Intermediate	https://github.com/AIRMEC/HECTOR	Partially available
Survival prediction	Li *et al*. (2023) [76]	Glioblastoma, glioma, gastric	WSI, CT; WSI, MRI	ViT	Weakly supervised	213 WSI–CT pairs; 365 WSI–MRI pairs	Intermediate	No	No
Prognosis prediction	Chen *et al*. (2022) [14]	Glioma, CCRCC	WSI, SNP, RNA-Seq, CNV	CNN, GNN, SNN	Supervised	1186 patients	Intermediate	https://github.com/mahmoodlab/PathomicFusion	Yes

*Note*: CRC, colorectal cancer; CNN, convolutional neural network; GNN, graph neural network; IMC, imaging mass cytometry; NSCLC, non–small cell lung cancer; DLBCL, diffuse large B-cell lymphoma; LSTM, long short-term memory; WSI, whole slide image; ViT, vision transformer; CCRCC, clear cell renal cell carcinoma; SNN, self-normalizing networks.

In certain cancers, such as lymphoma, predicting interim outcomes is vital for adjusting therapeutic regimens and improving quality of life. Cheng *et al*. proposed a multimodal approach based on PET-CT that employs a contrastive hybrid learning strategy to identify primary treatment failure (PTF) in diffuse large B-cell lymphoma (DLBCL), providing a noninvasive tool for assessing PTF risk [53]. Distant recurrence significantly contributes to poor prognosis in cancer patients, yet predicting this risk remains challenging despite insights into correlated factors. To this end, Volinsky-Fremond *et al*. designed a multimodal prognostic model that combines WSIs and tumor stage information to predict recurrence risk and assess the benefits of adjuvant chemotherapy in endometrial cancer, outperforming existing state-of-the-art (SOTA) methods. Their success can be attributed to the utilization of Vision Transformer (ViT) for representative learning of WSIs, alongside a three-arm architecture that integrates prognostic information from WSIs, molecular phenotypes predicted directly from WSIs, and tumor stage [29].

Combining MRI or CT with WSI enables a comprehensive analysis of patient prognosis from both macroscopic and microscopic perspectives. For instance, Li *et al*. presented a weakly supervised framework that employs a hierarchical radiology-guided co-attention mechanism to capture interactions between histopathological characteristics and radiological features, facilitating the identification of prognostic biomarkers with multimodal interpretability [76]. Chen *et al*. calculated the Kronecker product of unimodal feature representations to encode pairwise feature communications across modalities, controlling each modality’s contribution through a gating-based attention mechanism, thereby yielding an end-to-end framework that combines histological and genomic data for survival outcome prediction [14].

In summary, patient survival is influenced by numerous factors, and the intricate interplay among these variables poses significant challenges for prognostic accuracy, even with extensive clinical data on tumors. MDL presents a novel method for integrating diverse indicators related to tumor prognosis, offering the potential to discover new prognostic biomarkers from cross-scale data. Recent models employing attention mechanisms have enhanced the interpretability of multimodal features, driving advancements in AI applications for clinical use.

#### Treatment decision and response monitoring

Neoadjuvant chemotherapy, targeted therapy, and immunotherapy are increasingly integral to cancer management. The modern demand for more effective treatments underscores the need for accurate, personalized tests over one-size-fits-all approaches. For example, programmed death ligand-1 (PD-L1) expression status, evaluated via immunohistochemistry (IHC), serves as a clinical decision-making tool for immune checkpoint blockade (ICB) therapy. However, many treatments lack specific clinical indicators, highlighting an urgent need to identify biomarkers that can predict treatment benefits and support personalized therapy. This review examines recent advancements in MDL-based treatment decision-making and response monitoring (Table 5).

**Table 5:** Representative studies focus on treatment decision and response prediction

Topic	Study (year)	Cancer	Modality	Backbone	Learning method	Dataset	Fusion strategy	Code availability	Data availability
Treatment decision	Ma *et al*. (2023) [79]	Intramedullary glioma	MRI, clinical data	Transformer, DNN	Supervised	461 patients	Early	No	Need request
Treatment decision	Huang *et al*. (2022) [30]	CRC	WSI, clinical data	CNN, MLP	Supervised	509 patients	Early	https://github.com/hkmgeneis/MMDL/tree/master	Yes
Treatment decision	Esteva *et al*. (2022) [80]	Prostate	WSI, clinical data	CNN	Self-supervised	5654 patients	Early	Need request	Need request
Treatment decision	Zhou *et al*. (2023) [81]	Nasopharyngeal	MRI, CT	GAN	Supervised	50 patients	Intermediate	No	No
Response prediction	Joo *et al*. (2021) [82]	Breast	MRI, clinical data	3D CNN	Unsupervised	536 patients	Early	No	No
Response prediction	Zhou *et al*. (2023) [83]	CRC liver metastases	IHC scores, PET-CT, clinical	CNN	Unsupervised	307 patients	Intermediate	No	Need request
Response prediction	Gu *et al*. (2024) [84]	Breast	Ultrasound, clinical data	CNN	Supervised	170 patients	Early	Need request	Need request
Response prediction	Rabinovici-Cohen *et al*. (2022) [85]	Breast	MRI, IHC, clinical data	CNN	Unsupervised	1738 patients	Early, late	No	No

*Note*: DNN, deep neural network; WSI, whole slide image; CNN, convolutional neural network; MLP, multiple layer perceptron; GAN, generative adversarial network; CRC, colorectal cancer; IHC, immunohistochemistry.

To establish appropriate treatment paradigms and prognostic assessments for intramedullary gliomas, Ma *et al*. employed a Swin Transformer to segment lesions from multimodal MRI data. They combined extracted radiomic features with clinical baseline data to predict tumor grade and molecular phenotype [79]. Tumor mutational burden (TMB) has emerged as a promising indicator of the efficacy and prognosis of ICB therapy in tumors. Huang *et al*. developed a surrogate method for predicting TMB from WSIs in CRC by training a multimodal model that incorporates WSIs alongside relevant clinical data [30]. Esteva *et al*. created an integration framework for histopathology and clinical data to predict clinically relevant outcomes in prostate cancer patients, demonstrating enhanced prognostic accuracy compared to existing tools and providing evidence for treatment personalization [80]. Zhou *et al*. introduced a cascade multimodal synchronous generation network for MRI-guided radiation therapy, optimizing time and costs by generating intermediate multimodal sMRI and sCT data, incorporating attention modules for multilevel feature fusion [81].

Estimating the efficacy of therapeutic approaches is equally crucial. While some cancer patients may experience significant improvement during early targeted therapy, resistance can develop, rendering treatment ineffective and exposing patients to adverse side effects. Given the variability and dynamic nature of treatment responses, current research focuses on developing effective prediction methods, particularly noninvasive approaches. Pathologic complete response (pCR) is a recognized metric for evaluating the efficacy of neoadjuvant chemotherapy and serves as an indicator of disease-free survival and OS. Joo *et al*. developed a fusion model integrating clinical parameters and pretreatment MRI data to predict pCR in breast cancer, outperforming unimodal models [82]. Zhou *et al*. combined PET-CT, clinical variables, and IHC scores within a multimodal framework to predict the efficacy of bevacizumab in advanced CRC patients, utilizing a 2.5D architecture for feature extraction [83]. Gu *et al*. applied a DenseNet-121-based multimodal framework to integrate ultrasound and clinicopathological data for stratifying responses to neoadjuvant therapy in breast cancer [84]. Rabinovici-Cohen *et al*. predicted post-treatment recurrence in breast cancer patients by combining MRIs, IHC markers, and clinical data within a heterogeneous multimodal framework, demonstrating the advantages of multimodal fusion [85].

Accurate prediction of treatment outcomes before, during, and after therapy is essential for developing optimal individualized strategies, ultimately enhancing progression-free survival and OS for cancer patients. Current MDL methodologies exhibit remarkable advantages in integrating multisource data, often surpassing single-modality models in accuracy, positioning them favorably to advance clinical decision-making and efficacy evaluation in oncology.

## Discussion and conclusion

Recent advancements in medical imaging and sequencing technologies have led to an exponential increase in biomedical multimodal data. As the demand for precise tumor diagnosis and personalized treatment continues to rise, effectively harnessing this wealth of data presents a significant challenge in clinical oncology. The impressive success of DL in domains such as computer vision and NLP has catalyzed its application in tumor diagnosis and treatment within medical AI. Substantial evidence indicates that multimodal fusion modeling of biomedical data outperforms single-modality approaches in performance metrics [70, 78, 82, 85]. Consequently, we propose that MDL methods have the potential to serve as powerful tools for integrating multidisciplinary diagnostic and therapeutic data in oncology.

This paper first introduces the key data modalities relevant to clinical tumor management (Supplementary Materials 1), discussing their clinical significance. We also provide a summary of publicly available multimodal oncology datasets, ranging from large-scale databases like TCGA and TCIA to specialized datasets, such as HAM10000, which focus on specific tumor types or populations. These resources offer valuable data for researchers in the field.

We then outline fundamental DL concepts and common network architectures (Supplementary Materials 2), guiding researchers in selecting appropriate frameworks and methods for constructing MDL models. A review of SOTA modality-specific and multimodal representation techniques follows, with a focus on early, intermediate, and late fusion strategies. Evidence suggests that intermediate fusion models often outperform early or late fusion approaches [55, 68], so we provide an in-depth discussion of this method, categorizing it into homogeneous and heterogeneous fusion, as well as marginal versus joint fusion. This categorization allows readers to choose suitable representation and fusion strategies based on the heterogeneity and computational demands of their multimodal data.

Finally, we explore cutting-edge applications of MDL in oncology, covering areas such as multimodal data processing, tumor detection and diagnosis, prognosis prediction, treatment selection, and response monitoring. These applications highlight the latest advancements and emerging trends in MDL for precision oncology. However, challenges remain, as detailed in the following part.

### Challenge 1: scarcity of large open-source multimodal datasets and annotated information

Stringent ethical reviews constrain the acquisition of medical data, leading to a shortage of multimodal datasets, which contradicts AI’s reliance on big data. To improve model generalization, training on multicenter datasets is often necessary, but privacy concerns and labor-intensive data collection methods impede data sharing. FL has emerged as a promising solution, allowing for distributed model training without direct data exchange. [86]. In FL, only model parameters are shared and aggregated, addressing data privacy issues. As FL technology evolves, real-time data circulation among medical centers will become more feasible, enabling large-scale, standardized biomedical multimodal datasets.

Another common issue with multimodal datasets is modality incompleteness. For example, full multimodal MRIs typically consist of pre-contrast T1, T2, fluid attenuated inversion recovery, and post-contrast T1 scans. Missing sequences due to factors such as acquisition protocols, scanner availability, or patient-specific issues complicate joint modeling. In practice, prioritizing modality completeness or diversity depends on the task; for instance, when crucial modalities are missing, completeness should take precedence, whereas modality diversity may be more beneficial in other cases. Approaches to address missing modalities include modality synthesis, knowledge distillation, latent feature models, and domain adaptation techniques [87–89]. However, challenges such as long training times and model complexity remain, underscoring the need for more efficient solutions. To enhance clinical applicability, we recommend prioritizing clinically accessible and affordable modalities over complex datasets, enabling broader adoption of multimodal AI in precision oncology.

Furthermore, the scarcity of fully annotated multimodal datasets remains a bottleneck for MDL model development. While vast amounts of unlabeled cross-modal data are available, labeled data are limited and often noisy. Improving annotation reliability through partial label information is critical. Our review identifies two weakly supervised annotation approaches: (1) active learning, which selects reliable labels from pseudo-clusters and iterates from “easy” to “hard” annotations, and (2) data- and knowledge-driven annotation, which enhances accuracy by leveraging data characteristics and prior knowledge. These approaches can improve annotation efficiency and model robustness, advancing the application of MDL in precision oncology.

### Challenge 2: insufficient fine-grained modeling in MDL and the need for model optimization

Precision oncology is a multifaceted process involving various stages, and the increasing complexity of new therapies continues to challenge AI applications in oncology. While current MDL efforts focus on tasks such as tumor segmentation, detection, diagnosis, prognosis, and treatment decision support, many areas remain underexplored, and fine-grained models are often lacking. To improve generalization across tasks, it is essential to integrate diverse techniques and domain-specific expertise, enhancing the capability of pretrained models.

The high heterogeneity and cross-scale nature of multimodal medical data pose significant challenges for efficient integration. Fusion strategies are typically categorized into early, intermediate, and late fusion. Early fusion, while intuitive, often fails to establish deep interactions between modalities, leading to suboptimal information utilization. Intermediate fusion generates more diverse fused features but increases model complexity, which can lead to overfitting. Late fusion, typically used at the decision level as an ensemble method, becomes less efficient as the number of modalities grows, resulting in linear increases in parameter count, training inefficiency, and greater sensitivity to modality noise.

Overall, existing methods struggle to balance intra-modality processing with inter-modality fusion, resulting in performance bottlenecks and increased computational costs. Promising solutions include compressing multimodal architectures, such as multitask models that can train on diverse data types (e.g. images, videos, and audio) simultaneously. Hybrid fusion approaches, which combine the strengths of different fusion strategies, also hold potential. However, the effectiveness of these models, originally designed for natural images or audiovisual data, remains to be fully validated in the context of biomedical data.

### Challenge 3: poor interpretability of MDL

Explainability has become a major concern in medical AI. The high dimensionality and heterogeneity of multimodal data exacerbate this issue, and the latent embeddings generated after data fusion frequently lack clear connections to the original modalities, further hindering transparency.

Current efforts to enhance explainability typically leverage domain knowledge. Techniques such as ablation studies, feature clustering, and activation maps illuminate key decision areas, helping researchers and clinicians better understand the decision-making processes [90–92]. Some studies have explored graph-based methods, particularly in radiomics and omics, to illustrate relationships between data components and offer more intuitive explanations [93]. Model-agnostic methods, such as Local Interpretable Model-Agnostic Explanations, approximate complex model behaviors with simpler local models to improve interpretability [94].

Debate continues within the academic community about whether AI models should inherently possess explainability or rely on *post hoc* interpretability techniques (e.g. saliency maps or attention mechanisms). Future AI applications should prioritize biologically inspired explainable models that enhance performance while providing clear, understandable rationales for their decisions, thereby fostering clinician trust. Incorporating clinical domain knowledge into model design and developing user-friendly interaction platforms can help mitigate the “black-box” nature of AI tools, ultimately improving diagnostic accuracy. Additionally, better visualization tools will be essential for addressing interpretability challenges. These tools will visually represent the internal workings of models, making it easier for users to grasp decision rationales.

### Challenge 4: static models and group spectroscopy in MDL

Current MDL models are often static, resulting in delays in tumor prediction and hindering timely assessments of tumor progression, drug resistance, and toxicity. Future models should incorporate dynamic medical domain knowledge, establishing real-time MDL frameworks to improve data processing and integration. Additionally, mechanisms to manage modality inconsistency and missing data would broaden the applicability of these models.

While most existing models are group based, precision oncology requires personalized treatment plans tailored to individual patients. Therefore, patient-specific MDL models are a critical future direction. Other challenges include the evaluation of MDL models in clinical context, and the security of multimodal data collection, transmission, and sharing. Multidisciplinary collaboration are needed to solve these issues.

### Challenge 5: evaluation of MDL models in clinical settings

Evaluating the clinical effectiveness of MDL models presents a novel and formidable challenge. Due to the inherent complexity of MDL architectures, the high heterogeneity and dimensionality of the data, and the diverse nature of the modalities involved, clinical assessment goes beyond simply measuring model accuracy. It must also account for time and data costs, as well as the incremental information gain provided by the inclusion of additional modalities. These factors are critical for optimizing cost-effectiveness and harnessing the synergistic potential of multimodal data. Consequently, in addition to conventional evaluation metrics used for unimodal models, it is important to incorporate indicators such as modality-specific information gain (e.g. mutual information), clinical feasibility (as assessed by multidisciplinary expert panels), and the computational complexity that escalates with the increasing number of modalities (e.g. linear or exponential growth). As MDL methods continue to evolve and see broader clinical adoption, establishing a rigorous evaluation framework will be indispensable.

In summary, our research highlights the growing role of MDL in precision oncology, fueled by the rapid expansion of biomedical big data and advancements in DL. Multimodal fusion methods offer substantial value for cancer management by integrating diverse modalities to provide comprehensive and accurate insights. However, the full potential of multimodal data remains underexplored. Key improvements are needed in handling data heterogeneity, refining fusion strategies, and optimizing network architectures for clinical scenarios.

**Key Points**

- This review synthesizes recent advances in MDL for precision oncology, covering applications in image processing, diagnosis, prognosis prediction, treatment decisions, and response monitoring. We also discuss publicly available multimodal datasets and provide a comparative analysis of deep learning techniques, modality representation, and fusion strategies.
- While multimodal models generally show improved performance over unimodal ones, attention-based architectures and intermediate fusion strategies are often effective. However, with varied data noise, evaluation metrics, and statistical methods between studies, definitive conclusions on the superiority of specific methods are not yet established.
- Despite progress, challenges in integrating multimodal data persist. Effective fusion methods and adaptive MDL frameworks are crucial to overcoming issues like data heterogeneity, incompleteness, and feature redundancy, paving the way for the broader adoption of precision oncology.

## Supplementary Material

Supplementary_materials_bbae699

## Data Availability

Details about the data discussed in this study have been incorporated in the article. No additional data were generated for this study.

## Notes

Huan Yang is Associate Professor of Yangtze Delta Region Institute (Quzhou) at University of Electronic Science and Technology of China. Her research engages a range of issues in computational methods for cancer diagnosis and prognosis.
