# Supplementary material for: Multimodal deep learning approaches for precision oncology: a comprehensive review
Source: Brief Bioinform. 2025 Jan 5;26(1):bbae699. doi: 10.1093/bib/bbae699 (PMC11700660; doi:10.1093/bib/bbae699)
Supplement: Supplementary_materials_bbae699 [file supplementary_materials_bbae699.docx]

**Supplementary File**

**Contents**

[Materials 1: overview of clinical oncology data modalities 2](#_Toc19297)

[Radiomics 2](#_Toc7565)

[X-ray 2](#_Toc2460)

[Computed tomography (CT) 2](#_Toc16037)

[Magnetic resonance imaging (MRI) 2](#_Toc4378)

[PET-CT and PET-MRI 3](#_Toc28031)

[Pathomics 3](#_Toc9146)

[Ultrasonography 4](#_Toc2745)

[Endoscopy 4](#_Toc13527)

[Omics 5](#_Toc25754)

[Clinical data 6](#_Toc10414)

[Multiplexed images 6](#_Toc2378)

[Dermoscopy 7](#_Toc11291)

[Materials 2: prevalent architectures of DL models 8](#_Toc21544)

[Convolutional neural network (CNN) 8](#_Toc20269)

[Fully convolutional network (FCN) 8](#_Toc22156)

[Recurrent neural network (RNN) 8](#_Toc8538)

[Transformer 9](#_Toc32675)

[AutoEncoder (AE) 10](#_Toc17501)

[Graph neural network (GNN) 10](#_Toc17500)

[Materials 3: abbreviations 11](#_Toc21256)

[References 14](#_Toc20342)

## Materials 1: o**verview of clinical oncology data modalities**

A comprehensive understanding of oncology data type’s sources, characteristics, and common applications is crucial for developing effective multimodal fusion models.

**Radiomics**

Imaging plays an irreplaceable role in cancer clinical programs, providing structural, morphological, metabolic, and functional insights. Radiomics refers to the extraction of non-invasive, reproducible, and quantitatively derived features from medical images, which reflect underlying pathophysiological processes. This typically involves the analysis of imaging modalities such as X-rays, CT, PET, MRI, PET-CT, or PET-MRI.

**X-ray**

X-ray imaging relies on the differential density and thickness of human tissues, producing black-and-white images with varying brightness and contrast. Digital radiography (DR) has reduced radiation exposure, improved image reconstruction speed, and enhanced image quality [1]. Recently, contrast-enhanced spectral mammography (CESM) has improved sensitivity and diagnostic specificity for early breast cancer detection [2].

**Computed tomography (CT)**

CT imaging provides detailed cross-sectional images, playing a crucial role in early tumor detection, diagnosis, treatment planning, and prognosis. Contrast-enhanced CT (CECT) enhances tissue visibility, with phases including precontrast, arterial, and portal venous [3]. Key advantages of CT include non-invasiveness, speed, convenience, and high resolution. However, limitations include ionizing radiation exposure and potential contrast agent side effects [4].

**Magnetic resonance imaging (MRI)**

MRI uses magnetic fields and radiofrequency waves, offering a safer alternative for children, pregnant women, and patients requiring frequent imaging [5]. Its superior soft-tissue contrast and broad availability make it ideal for imaging the brain, spinal cord, joints, and vascular structures. MRI can acquire images in multiple planes (axial, sagittal, coronal) and assess metabolic activity using functional imaging techniques such as functional MRI (fMRI) and diffusion-weighted imaging (DWI) [6]. MRI's main limitations include long scan times, contraindications [7], and issues such as high costs, potential sedation side effects, image corruption, and limited scanner availability [8].

**PET-CT and PET-MRI**

Positron emission tomography (PET) is a major advancement in molecular imaging. Initially, it was primarily employed as a research tool for functional brain imaging. Nowadays, with the development of hybrid imaging technologies, PET has been combined with computed tomography (PET-CT) or magnetic resonance imaging (PET-MRI) and has become a commonly used tool in clinical oncology.

The integration of PET and CT was achieved by minimally modifying and combining the two subsystems into a single gantry, allowing for the sequential acquisition of both imaging modalities. PET-CT can detect biochemical and physiological abnormalities related to diseases before anatomical changes occur, hence widely used for detecting tumor metastases, measuring tumor volume, and evaluating treatment efficacy. Generally, PET images have lower resolution than CT or MRI, approximately 3-5 millimeters [9]. Additionally, PET images are susceptible to noise from limited photon counts. Fortunately, advances in crystal detector technologies and image reconstruction techniques have shown promise to improve PET image quality and broaden its applications [10]. Still, there are concerns regarding comparatively high dose of radiation and expensive examination expenses.

PET-MRI integrates the molecular imaging capabilities of PET with the superior soft tissue contrast and multiparametric imaging features of MRI. Over the past two decades, the introduction of MR-compatible photodetectors (e.g. APDs and SiPMs) has leaded the development of various clinical PET-MRI systems [11]. Currently, DL has shown promising applications in PET-MRI, including segmentation, reconstruction, and outcome modeling [12-14]. Despite these advancements, further optimization of instrumentation and simplification of clinical workflows are necessary. Major challenges include limitations in MRI-guided attenuation correction, particularly in whole-body imaging, and establishing a clear clinical role for this modality to justify the additional investment cost.

**Pathomics**

Pathologists' evaluation of histopathological slides remains the gold standard for tumor diagnosis. Hematoxylin and eosin (H&E)-stained tissue sections provide an accessible and informative data source [15]. With the widespread adoption of whole-slide scanners, most glass slides can be digitized into whole slide images (WSI) for storage and computer-aided analysis [16, 17]. Moreover, linking H&E images with the genome using AI algorithms is increasingly becoming a reality [18].

For poorly differentiated tumors or those classified as not otherwise specified (NOS) diagnoses, immunohistochemical (IHC) staining is needed in addition to routine H&E staining [19]. By evaluating the expression of specific molecular markers (e.g., EGFR, KRAS, etc.), IHC can offer personalized treatment strategy suggestions for cancer patients, as exemplified by the selection of targeted therapies and immunotherapies. However, the diagnosis process is relatively time-consuming and expensive [20, 21]. Recently, stain transformation methods have offered cost-effective and efficient alternatives for patients with limited tissue samples or medical budgets, though accuracy still requires improvement [22].

**Ultrasonography**

Ultrasound imaging is an acoustic method that uses high-frequency sound waves to visualize internal structures. Based on different imaging modes, ultrasound imaging can be divided into grayscale 2D ultrasound, ultrasound elastography (such as strain elastography and shear wave elastography), and contrast-enhanced ultrasound (CEUS). Ultrasound scanning has a broad range of applications in tumor diagnosis and treatment. For example, conventional ultrasound (CUS) is the first-line diagnostic tool for patients with metastatic cervical lymphadenopathy (CLA) [23]. Ultrasound elastography (UE) and CEUS are widely used for breast cancer screening [24]. Ultrasound is also a routine diagnostic method for thyroid cancer, where shear wave elastography (SWE) and color Doppler ultrasound (CDUS) are ordered as auxiliary examinations [25, 26].

Ultrasoundgraphy is more suitable for soft tissue imaging and has advantages, including being non-invasive, easy to operate, radiation-free, real-time imaging, low cost, and widely accessible. However, the image resolution is relatively low; image quality is susceptible to body size, fat, and gas; the technique heavily dependent on expertise. Additionally, ultrasound has limited specificity in distinguishing between benign and malignant masses, often necessitating the use of complementary imaging modalities and additional tests for a complete evaluation.

**Endoscopy**

Endoscopic imaging uses an optical system to capture images of internal body cavities or organs. Depending on the imaging method, endoscopes can be classified into white-light endoscopy, chromoendoscopy, image-enhanced endoscopy (IEE), and probe-based confocal laser endomicroscopy (pCLE) [27]. Endoscopic examinations are commonly used for cancer screening, diagnosis, minimally invasive treatment, monitoring, and follow-up in patients with gastrointestinal, respiratory, or urinary symptoms. For instance, colonoscopy is an essential method for detecting colorectal lesions, where white-light endoscopy assesses lesion morphology and IEE evaluates the surface and vascular status of the intestines [28]. Digestive endoscopy is the primary method for identifying early gastric cancer [29].

Endoscopy provides real-time high-resolution imaging of internal organ surfaces, aids in biopsy sampling for definitive diagnosis, and enables minimally invasive procedures such as polyp removal, hemostasis, and foreign body extraction. However, as an invasive procedure, endoscopy is limited by patient conditions, with many contraindications and major risks. Moreover, endoscopy is not suitable for detection of deep tissue lesions. Small, early, or complex lesions may be missed or inadequately sampled, increasing the risk of misdiagnosis.

**Omics**

Recent advancements in high-throughput sequencing technologies have generated multiple omics data, including genomics, transcriptomics, epigenomics, and proteomics. Integrating these multi-omics datasets enhances understanding of oncology in ways unattainable through single-omics data alone.

Genomics involves the study of the entire genome sequence, with gene (DNA) sequencing technology evolving through Sanger sequencing, high-throughput sequencing, and single-molecule sequencing—referred to as first, second (next), and third-generation sequencing, respectively. In oncology, genomics applications such as whole-genome analysis, cancer susceptibility gene testing, and driver gene mutation detection, providing the foundation for precision therapies [30, 31].

Transcriptomics systematically studies the transcriptional profiles at the overall transcription level to reveal the molecular mechanisms of complex biological pathways and regulatory networks. Transcriptome sequencing (RNA-Seq) uses high-throughput sequencing technology to analyze the mRNA, rRNA, tRNA, small RNA, and non-coding RNA present in cells or tissues at specific time points [32]. This approach allows researchers to study transcript changes in relation to temporal, spatial, tissue, and sample variations [33].

Epigenetics studies genetic information involving changes other than nucleotide sequences, such as DNA methylation, RNA methylation, gene imprinting, histone covalent modifications, and chromatin remodeling [34]. Common sequencing technologies include Whole Genome Bisulfite Sequencing (WGBS) and ChIP-Seq. Research has demonstrated that abnormal DNA methylation patterns and histone modifications are associated with various cancers, such as CRC and ovarian cancer [35, 36]. Epigenomic analysis plays a vital role in understanding gene regulatory mechanisms involved in tumorigenesis, leading to the discovery of new therapeutic targets and the development of epigenetic drugs.

Proteomics explores protein expression, post-translational modifications, and interactions of all proteins within cells, offering a comprehensive understanding of disease mechanisms and cellular metabolism at the protein level. Proteomics technologies primarily rely on liquid chromatography-tandem mass spectrometry (LC-MS/MS) and protein microarrays [37, 38]. Proteomics facilitates the discovery of tumor biomarkers, drug target identification, and analysis of protein interaction networks, providing more concrete information on tumor microenvironment and signal transduction in tumor cell [39].

With advances in sequencing technologies and decreasing costs, the exponential growth of clinical oncology multiomics data is poised to uncover novel biological insights and mechanisms of tumor pathogenesis, thus enhancing our understanding of cancer progression and prognosis.

**Clinical data**

Clinical information are the most fundamental data accompanying patients throughout the entire diagnostic and therapeutic cycle. These include demographic information, general measures of health status, laboratory biochemical test results, imaging reports, pathological data, surgery-related data, and therapy-related data. Clinical data are extensive in both content and scale. For instance, sex and age greatly influence cancer incidence and prognosis [40]. Body mass index (BMI) is associated with the risk of various common adult cancers [41]. Liver and kidney function variables are closely linked to surgical and medication risks [42]. Clinical information is typically stored in the form of EHRs. Combining clinical information with imaging, molecular omics, and pathology enables the assessment of tumor characteristic across multiple scales and dimensions at lower costs. However, potential risks exist, including high subjectivity of physicians, entry errors, and inaccuracies in patient self-reported data.

**Multiplexed images**

Multiplexed imaging integrates multiple imaging and sequencing technologies, providing high-dimensional and high-resolution image information that single modalities cannot achieve. These technologies include Imaging Mass Cytometry (IMC), Imaging Mass Spectrometry (IMS), single-cell RNA sequencing (scRNA-seq), and spatial transcriptomics (ST) techniques, etc. IMC employs isotope-tagged antibodies to capture spatial expression maps of up to 30 biomarkers at the single-cell level [43]. IMS is a chemical modality used in molecular pathology that augments histopathological investigations with rich spatio-molecular information [44]. ScRNA-seq enables the large-scale whole-transcriptome profiling of single cells in a cell population [45]. ST positions tissue sections onto spatially barcoded arrays at 50-micron resolution that allowing deep understanding of how gene expression is connected to the spatial organization of cells and molecules [46]. Notably, multiplexed imaging aids in the foundation of personalized medicine. However, the high cost of equipment and reagents, technical complexity, and the need for improved standardization present significant challenges.

**Dermoscopy**

Dermoscopy imaging utilizes optical magnification and skin translucency techniques, offering valuable morphological information that facilitates the early detection and diagnosis of skin cancers, including melanoma, basal cell carcinoma, cutaneous squamous cell carcinoma, etc [47]. Main advantages of dermoscopy are non-invasiveness, ease of operation, real-time imaging, and high resolution. However, dermoscopy imaging also has limitations, such as susceptibility to lighting conditions and skin types. Diagnostic accuracy also depends heavily on the physician's experience and skill level, and the specificity for different lesions is relatively low.

To summarize, the diversity and heterogeneity of multimodal clinical oncology data provide a plentiful information sea for precision medicine. In the following section, we will overview public multimodal oncology data, and state-of-the-art (SOTA) DL techniques, as well as neural network architectures, which jointly lay the foundation of multimodal fusion.

## Materials 2: p**revalent architectures of DL models**

Neural networks have revolutionized tumor research by facilitating the integration of diverse data modalities. This section examines prevalent DL architectures that applicable across diverse data types.

**Convolutional neural network (CNN)**

CNNs are a class of feedforward neural networks distinguished by local connectivity and weight sharing, making them particularly effective for image and video analysis. They process pixel intensities through convolution operations, hierarchically extracting features at multiple scales. Each convolutional layer consists of various filters (kernels) that traverse the input image to produce feature maps. Neurons in these maps connect to a localized region in the previous layer, corresponding to the filter size, known as the receptive field. The weight-sharing mechanism applies identical filters across the entire image, reducing parameter counts and enabling feature detection throughout the image. In oncology, CNNs are widely employed for lesion detection, segmentation, and classification [48-50]. Notable CNN architectures in this domain include VGG, ResNet, Inception, and EfficientNet.

**Fully convolutional network (FCN)**

FCNs consist of fully convolutional and deconvolutional components. The fully convolutional segment utilizes classic CNN structures, such as ResNet, for feature extraction, while the deconvolutional component employs upsampling to restore the original image dimensions, compensating for reductions caused by convolution and pooling. By replacing fully connected layers with convolutional layers, FCNs generate heatmap outputs rather than discrete classes, accommodating inputs of arbitrary size while maintaining consistent output dimensions. Skip connections across varying depths enhance FCNs' robustness and accuracy, rendering them particularly suitable for tumor lesion segmentation. U-Net is a prominent FCN architecture utilized for this purpose [51].

**Recurrent neural network (RNN)**

RNNs are specifically designed for sequential data processing through cyclic architectures. They recursively compute outputs while establishing dependencies between current outputs and prior states through interconnected nodes forming closed loops. However, they encounter challenges such as gradient explosion and vanishing. To mitigate these issues, several architectures have emerged, including Bidirectional RNNs (BRNNs), Long Short-Term Memory (LSTM) networks , and Gated Recurrent Units (GRUs). When integrated with CNNs, RNNs can tackle computer vision challenges. In oncology, RNNs excel at analyzing genomic sequences and EHRs [52]. Although RNNs theoretically handle sequences of arbitrary length, practical implementations often constrain state influences to a finite number of preceding states to optimize computational efficiency. Moreover, training RNNs can be resource-intensive, prompting the use of transfer learning techniques to mitigate costs.

**Transformer**

Transformers have achieved remarkable success in NLP. A standard Transformer consists of an encoder and a decoder, incorporating key components such as input embeddings, positional encodings, residual connections, layer normalization, multi-head attention, and feed-forward networks [53]. The multi-head attention mechanism enables the model to focus on different segments of the input sequence, facilitating parallel processing. Unlike RNNs, Transformers excel in parallelization and effectively capture long-range dependencies. Moreover, their architecture is highly adaptable: they can function as an encoder (e.g., Bidirectional Encoder Representations from Transformers, or BERT), a decoder (e.g., Generative Pretrained Transformer, or GPT), or as an encoder-decoder (e.g., BigBird), allowing for diverse sequence analysis tasks [54-56]. The effectiveness of Transformers is particularly evident in healthcare applications, including clinical trial matching, treatment recommendations, and laboratory chatbots [57, 58].

To overcome the limitations of Transformers with image data, the Google Brain team introduced the Vision Transformer (ViT) model, which utilizes a self-attention mechanism to analyze image features, challenging the traditional dominance of CNNs. ViT shows significant potential in enhancing the accuracy of diagnostic and prognostic tasks, such as tumor subtyping and patient outcome prediction [59].

A distinctive feature of the attention model, compared to CNNs and RNNs, is its explicit representation of the varying contributions of different input data segments as trainable parameters, enhancing clinical interpretability. For example, the model can highlight the importance of specific areas in slides for biomarker prediction [60]. However, both Transformer and ViT models require extensive pre-training on large datasets, and fine-tuning on private datasets can be time-consuming. When processing high-resolution images, such as WSIs, ViT incurs significant computational costs due to patch size constraints. Future research will likely focus on reducing these costs and improving computational efficiency, as well as developing Transformer variants that perform effectively with small sample sizes

**AutoEncoder (AE)**

AEs are prominent unsupervised neural networks that consist of an encoder and a decoder. The encoder compresses input data into a low-dimensional representation, while the decoder reconstructs the original data from this encoding. Through iterative training, the model's output aligns progressively with the input, effectively capturing essential data features and achieving dimensionality reduction. Variants of AEs include stacked AE, deep AE, Denoising AE, sparse AE, and variational AE (VAE) [61]. Nevertheless, AEs are prone to overfitting, and their effectiveness often depends on the chosen architecture and hyperparameters.

**Graph neural network (GNN)**

GNNs extend traditional neural networks to effectively process graph-structured data, showcasing exceptional performance in biomedical applications, particularly in gene expression prediction, molecular interaction prediction, and protein structure prediction [62-64]. Notable GNN architectures include Graph Convolutional Networks (GCNs), Gated Graph Neural Networks (GGNNs), and Graph Attention Networks (GANs) [65]. The selection of an appropriate GNN model should be informed by specific task requirements and data characteristics. For example, GCNs are particularly adept at handling non-Euclidean structured data, such as biomolecules and knowledge graphs, which lack translation invariance and feature variable numbers of neighboring nodes [66]. Given the increasing emphasis on the robustness and interpretability of DL methodologies, GNNs represent a promising avenue for the future of AI.

Despite their advantages, GNNs face limitations. Many GNN architectures are inherently shallow, constraining their ability to represent abstract features effectively. Dynamic graphs also pose challenges, as their structures evolve over time, complicating GNN adaptability. Additionally, scalability remains a significant hurdle, and graph generation algorithms often require considerable domain expertise. Advancements in adaptive methods for managing dynamic graphs and improving GNN scalability will further broaden their applicability to intricate graph-structured datasets.

Neural networks have revolutionized tumor research by facilitating the integration of diverse data modalities. The strategic selection of neural network architectures and training methodologies will empower researchers to significantly advance the development of highly efficient MDL fusion models.

## Materials 3: abbreviations

| **Abbreviation** | **Full name** |
| --- | --- |
| AE | AutoEncoder |
| AI | artificial intelligence |
| ASVM | Adaptive Support Vector Machine |
| BERT | Bidirectional Encoder Representations from Transformers |
| BMI | body mass index |
| B-mode | brightness-mode |
| BRNN | bidirectional RNN |
| CCA | Canonical Correlation Analysis |
| CDUS | color Doppler ultrasound |
| CECT | contrast-enhanced CT |
| CESM | contrast-enhanced spectral mammography |
| CEUS | contrast-enhanced ultrasound |
| CLA | cervical lymphadenopathy |
| CNN | convolutional neural network |
| CNV | copy number variation |
| Conv-LSTM | convolutional-long short time memory |
| CRC | colorectal cancer |
| CT | computed tomography |
| CUS | conventional ultrasound |
| DCIS | ductal carcinoma in situ |
| DELFI | DNA evaluation of fragments for early interception |
| DFS | disease-free survival |
| DL | deep learning |
| DLBCL | diffuse large B-cell lymphoma |
| DR | digital radiography |
| DWI | diffusion-weighted imaging |
| EHR | electronic health records |
| FCN | fully convolutional network |
| FL | federated Learning |
| FLAIR | fluid attenuated inversion recovery |
| fMRI | functional MRI |
| GAN | graph attention network |
| GCN | graph convolutional neural network |
| GGNN | gated graph neural network |
| GNN | graph neural network |
| GPT | generative pretrained Transformer |
| GRU | gated recurrent unit |
| H&E | hematoxylin and eosin |
| IARC | International Agency for Research on Cancer |
| ICB | immune checkpoint blockade |
| IEE | image-enhanced endoscopy |
| IHC | immunohistochemistry |
| IMC | imaging mass cytometry |
| IMGs | intramedullary gliomas |
| IMS | imaging mass spectrometry |
| LC-MS/MS | liquid chromatography-tandem mass spectrometry |
| LIME | Local Interpretable Model-Agnostic Explanations |
| LNM | lymph node metastasis |
| Lung-CLiP | Lung Cancer Likelihood in Plasma |
| MDL | multimodal DL |
| MIL | multiple instance learning |
| MRI | magnetic resonance imaging |
| MSI | mass spectrometry imaging |
| NLP | natural language processing |
| NOS | not otherwise specified |
| NSCLC | non-small cell lung cancer |
| ONM | occult nodal metastasis |
| OS | overall survival |
| pCLE | probe-based confocal laser endomicroscopy |
| pCR | pathologic complete response |
| PD-L1 | programmed death ligand-1 |
| PET | positron emission tomography |
| PTF | primary treatment failure |
| RNN | recurrent neural network |
| scRNA-seq | single-cell RNA sequencing |
| SE-mode | strain-elastography-mode |
| SL | supervised learning |
| SNV | single nucleotide variation |
| SOTA | state-of-the-art |
| SSL | self-supervised learning |
| ST | spatial transcriptomics |
| SUVmax | the maximum value of the standard uptake |
| SWE | shear wave elastography |
| TCGA | The Cancer Genome Atlas |
| TCIA | The Cancer Imaging Archive |
| TeUS | temporal enhanced ultrasound |
| TL | transfer learning |
| TMB | tumor mutational burden |
| TRUS | transrectal ultrasound |
| UE | ultrasound elastography |
| USL | unsupervised learning |
| VAE | variational AutoEncoder |
| ViT | vistion Transformer |
| WGBS | whole genome bisulfite sequencing |
| WSI | whole slide image |
| WSL | weakly supervised learning |

## References

1. Xu Y, Ma D, He W. Assessing the use of digital radiography and a real-time interactive pulmonary nodule analysis system for large population lung cancer screening, Eur J Radiol 2012;81:e451-456.

2. Nicosia L, Bozzini AC, Latronico A et al. Contrast-Enhanced Spectral Mammography: Importance of the Assessment of Breast Tumor Size, Korean J Radiol 2021;22:489-491.

3. Xie Z, Suo S, Zhang W et al. Prediction of high Ki-67 proliferation index of gastrointestinal stromal tumors based on CT at non-contrast-enhanced and different contrast-enhanced phases, European Radiology 2023;34:2223-2232.

4. Schmidt B, Saltybaeva N, Kolditz D et al. Assessment of patient dose from CT localizer radiographs, Medical Physics 2013;40:084301.

5. Semelka RC, Armao DM, Elias J et al. Imaging strategies to reduce the risk of radiation in CT studies, including selective substitution with MRI, Journal of Magnetic Resonance Imaging 2007;25:900-909.

6. Logothetis NK. What we can do and what we cannot do with fMRI, Nature 2008;453:869-878.

7. Panych LP, Madore B. The physics of MRI safety, J Magn Reson Imaging 2018;47:28-43.

8. Sharma A, Hamarneh G. Missing MRI Pulse Sequence Synthesis Using Multi-Modal Generative Adversarial Network, IEEE Trans Med Imaging 2020;39:1170-1183.

9. Maddahi J, Packard RR. Cardiac PET perfusion tracers: current status and future directions, Semin Nucl Med 2014;44:333-343.

10. Song TA, Chowdhury SR, Yang F et al. PET image super-resolution using generative adversarial networks, Neural Netw 2020;125:83-91.

11. Kim SY, Soh H, Jung JH et al. Direct and Indirect Chimeric Antigen Receptor T-Cell Imaging with PET/MRI in a Tumor Xenograft Model, Radiology 2024;310:e231406.

12. Choi JH, Kim HA, Kim W et al. Early prediction of neoadjuvant chemotherapy response for advanced breast cancer using PET/MRI image deep learning, Sci Rep 2020;10:21149.

13. Hayat H, Wang R, Sun A et al. Deep learning-enabled quantification of simultaneous PET/MRI for cell transplantation monitoring, iScience 2023;26:107083.

14. Rajagopal A, Natsuaki Y, Wangerin K et al. Synthetic PET via Domain Translation of 3-D MRI, IEEE Trans Radiat Plasma Med Sci 2023;7:333-343.

15. Fischer AH, Jacobson KA, Rose J et al. Hematoxylin and eosin staining of tissue and cell sections, Cold spring harbor protocols 2008;2008:pdb. prot4986.

16. Bera K, Schalper KA, Rimm DL et al. Artificial intelligence in digital pathology—new tools for diagnosis and precision oncology, Nature reviews Clinical oncology 2019;16:703-715.

17. Milam M, Koo C. The current status and future of FDA-approved artificial intelligence tools in chest radiology in the United States, Clinical Radiology 2023;78:115-122.

18. Schneider L, Laiouar-Pedari S, Kuntz S et al. Integration of deep learning-based image analysis and genomic data in cancer pathology: A systematic review, European journal of cancer 2022;160:80-91.

19. Okoye JO, Nnatuanya IN, Okoye J. Immunohistochemistry: a revolutionary technique in laboratory medicine, Clin Med Diagn 2015;5:60-69.

20. Yamabayashi S. Periodic acid—Schiff—Alcian Blue: A method for the differential staining of glycoproteins, The Histochemical Journal 1987;19:565-571.

21. Garvey W. Modified Elastic Tissue-Masson Trichrome Stain, Stain Technology 1984;59:213-216.

22. de Haan K, Zhang Y, Zuckerman JE et al. Deep learning-based transformation of H&E stained tissues into special stains, Nature communications 2021;12:1-13.

23. Giacomini CP, Jeffrey RB, Shin LK. Ultrasonographic evaluation of malignant and normal cervical lymph nodes. In: Seminars in Ultrasound, CT and MRI, 2013;236-247.

24. Fan Z, Gong P, Tang S et al. Joint localization and classification of breast masses on ultrasound images using an auxiliary attention-based framework, Med Image Anal 2023;90:102960.

25. Park AY, Kim J-A, Son EJ et al. Shear-wave elastography for papillary thyroid carcinoma can improve prediction of cervical lymph node metastasis, Annals of surgical oncology 2016;23:722-729.

26. Saller B, Moeller L, Görges R et al. Role of conventional ultrasound and color Doppler sonography in the diagnosis of medullary thyroid carcinoma, Experimental and clinical endocrinology & diabetes 2002;110:403-407.

27. Tajiri H, Niwa H. Proposal for a consensus terminology in endoscopy: how should different endoscopic imaging techniques be grouped and defined?, Endoscopy 2008;40:775-778.

28. Repici A, Badalamenti M, Maselli R et al. Efficacy of Real-Time Computer-Aided Detection of Colorectal Neoplasia in a Randomized Trial, Gastroenterology 2020;159:512-520.e517.

29. Pimentel-Nunes P, Libânio D, Marcos-Pinto R et al. Management of epithelial precancerous conditions and lesions in the stomach (MAPS II): European Society of Gastrointestinal Endoscopy (ESGE), European Helicobacter and Microbiota Study Group (EHMSG), European Society of Pathology (ESP), and Sociedade Portuguesa de Endoscopia Digestiva (SPED) guideline update 2019, Endoscopy 2019;51:365-388.

30. Wong M, Mayoh C, Lau LMS et al. Whole genome, transcriptome and methylome profiling enhances actionable target discovery in high-risk pediatric cancer, Nat Med 2020;26:1742-1753.

31. Li BT, Ross DS, Aisner DL et al. HER2 Amplification and HER2 Mutation Are Distinct Molecular Targets in Lung Cancers, J Thorac Oncol 2016;11:414-419.

32. Wang Z, Gerstein M, Snyder M. RNA-Seq: a revolutionary tool for transcriptomics, Nat Rev Genet 2009;10:57-63.

33. Peng J, Sun BF, Chen CY et al. Single-cell RNA-seq highlights intra-tumoral heterogeneity and malignant progression in pancreatic ductal adenocarcinoma, Cell Res 2019;29:725-738.

34. Recillas-Targa F. Cancer Epigenetics: An Overview, Arch Med Res 2022;53:732-740.

35. Yang X, Zhang S, He C et al. METTL14 suppresses proliferation and metastasis of colorectal cancer by down-regulating oncogenic long non-coding RNA XIST, Mol Cancer 2020;19:46.

36. Moschetta M, George A, Kaye SB et al. BRCA somatic mutations and epigenetic BRCA modifications in serous ovarian cancer, Ann Oncol 2016;27:1449-1455.

37. Broeckling CD, Beger RD, Cheng LL et al. Current Practices in LC-MS Untargeted Metabolomics: A Scoping Review on the Use of Pooled Quality Control Samples, Anal Chem 2023;95:18645-18654.

38. MacBeath G. Protein microarrays and proteomics, Nat Genet 2002;32 Suppl:526-532.

39. Savage SR, Yi X, Lei JT et al. Pan-cancer proteogenomics expands the landscape of therapeutic targets, Cell 2024;187:4389-4407.e4315.

40. Sung H, Ferlay J, Siegel RL et al. Global Cancer Statistics 2020: GLOBOCAN Estimates of Incidence and Mortality Worldwide for 36 Cancers in 185 Countries, CA Cancer J Clin 2021;71:209-249.

41. Li J, Chen Z, Wang Q et al. Microbial and metabolic profiles unveil mutualistic microbe-microbe interaction in obesity-related colorectal cancer, Cell Rep Med 2024;5:101429.

42. Kambakamba P, Slankamenac K, Tschuor C et al. Epidural analgesia and perioperative kidney function after major liver resection, Br J Surg 2015;102:805-812.

43. Ali HR, Jackson HW, Zanotelli VRT et al. Imaging mass cytometry and multiplatform genomics define the phenogenomic landscape of breast cancer, Nat Cancer 2020;1:163-175.

44. Casadonte R, Kriegsmann M, Kriegsmann K et al. Imaging Mass Spectrometry-Based Proteomic Analysis to Differentiate Melanocytic Nevi and Malignant Melanoma, Cancers (Basel) 2021;13:3197.

45. Zhang X, Li T, Liu F et al. Comparative Analysis of Droplet-Based Ultra-High-Throughput Single-Cell RNA-Seq Systems, Molecular Cell 2019;73:130-142.e135.

46. Castillo RL, Sidhu I, Dolgalev I et al. Spatial transcriptomics stratifies psoriatic disease severity by emergent cellular ecosystems, Sci Immunol 2023;8:eabq7991.

47. Yélamos O, Braun RP, Liopyris K et al. Dermoscopy and dermatopathology correlates of cutaneous neoplasms, J Am Acad Dermatol 2019;80:341-363.

48. Rasel MA, Abdul Kareem S, Kwan Z et al. Asymmetric lesion detection with geometric patterns and CNN-SVM classification, Comput Biol Med 2024;179:108851.

49. Wang W, Pan B, Ai Y et al. ParaCM-PNet: A CNN-tokenized MLP combined parallel dual pyramid network for prostate and prostate cancer segmentation in MRI, Comput Biol Med 2024;170:107999.

50. Esteva A, Kuprel B, Novoa RA et al. Dermatologist-level classification of skin cancer with deep neural networks, Nature 2017;542:115-118.

51. Huang YJ, Dou Q, Wang ZX et al. 3-D RoI-Aware U-Net for Accurate and Efficient Colorectal Tumor Segmentation, IEEE Trans Cybern 2021;51:5397-5408.

52. Ibrahim ZM, Bean D, Searle T et al. A Knowledge Distillation Ensemble Framework for Predicting Short- and Long-Term Hospitalization Outcomes From Electronic Health Records Data, IEEE J Biomed Health Inform 2022;26:423-435.

53. Vaswani A. Attention is all you need, arXiv preprint arXiv:1706.03762, 2017.

54. Devlin J. Bert: Pre-training of deep bidirectional transformers for language understanding, arXiv preprint arXiv:1810.04805, 2018.

55. Liu X, Zheng Y, Du Z et al. GPT understands, too, AI Open 2024;5:208-215.

56. Zaheer M, Guruganesh G, Dubey KA et al. Big bird: Transformers for longer sequences, Advances in Neural Information Processing Systems 2020;33:17283-17297.

57. Thirunavukarasu AJ, Ting DSJ, Elangovan K et al. Large language models in medicine, Nature Medicine 2023;29:1930-1940.

58. Yang HS, Wang F, Greenblatt MB et al. AI Chatbots in Clinical Laboratory Medicine: Foundations and Trends, Clin Chem 2023;69:1238-1246.

59. Han K, Wang Y, Chen H et al. A Survey on Vision Transformer, IEEE Trans Pattern Anal Mach Intell 2023;45:87-110.

60. Wagner SJ, Reisenbüchler D, West NP et al. Transformer-based biomarker prediction from colorectal cancer histology: A large-scale multicentric study, Cancer Cell 2023;41:1650-1661.e1654.

61. Zhai J, Zhang S, Chen J et al. Autoencoder and its various variants. In: 2018 IEEE international conference on systems, man, and cybernetics (SMC), 2018;415-419.

62. Zeng Y, Wei Z, Yu W et al. Spatial transcriptomics prediction from histology jointly through Transformer and graph neural networks, Brief Bioinform 2022;23:bbac297.

63. Wang G, Liu X, Wang K et al. Deep-learning-enabled protein-protein interaction analysis for prediction of SARS-CoV-2 infectivity and variant evolution, Nat Med 2023;29:2007-2018.

64. Li M, Cao Y, Liu X et al. Structure-Aware Graph Attention Diffusion Network for Protein-Ligand Binding Affinity Prediction, In: IEEE Trans Neural Netw Learn Syst, 2023;1-11.

65. Li R, Yuan X, Radfar M et al. Graph Signal Processing, Graph Neural Network and Graph Learning on Biological Data: A Systematic Review, IEEE Rev Biomed Eng 2023;16:109-135.

66. Zeng X, Gong J, Li W et al. Knowledge-driven multi-graph convolutional network for brain network analysis and potential biomarker discovery, Med Image Anal 2024;99:103368.
